# Strategies for adaptation of mAb-producing CHO cells to serum-free medium

**DOI:** 10.1186/1753-6561-5-S8-P112

**Published:** 2011-11-22

**Authors:** A Rita Costa, M Elisa Rodrigues, Mariana Henriques, Rosário Oliveira, Joana Azeredo

**Affiliations:** 1 IBB-Institute for Biotechnology and Bioengineering, Centre of Biological Engineering, University of Minho, Braga, Portugal

## Background

The large-scale production of biopharmaceuticals, such as monoclonal antibodies (mAbs), commonly requires the use of serum-free medium, for both safety and economical reasons. However, because serum is such an essential supplement of the growth medium of most mammalian cells, its removal demands a very time-consuming process of cell adaptation. In this process, cells are usually subjected to a gradual, step-wise, decrease of serum concentration in the medium. With the purpose of alleviating cell adaptation, other medium supplements such as insulin and trace elements can be used, either isolated or in combination. Thus, the aim of this study was to assess strategies for the adaptation of CHO cells to serum-free media, using different supplement combinations, as well as to identify the most critical steps of the process.

## Materials and methods

The study was divided in two experiments. In the first one, cultures of mAb-producing CHO-K1 cells were initiated in 24 well plates, using Dulbecco’s Modified Eagle Medium (DMEM) supplemented with 10% serum. The effect of five combinations of supplements that could support cells during adaptation was tested. These supplements included insulin and eight different trace elements. A methodology of gradual adaptation was followed, consisting of sequential steps of serum reduction. At the level of 0.625 % serum the medium was gradually switched from DMEM to the chemically-defined serum-free EX-CELL CHO DHFR^-^ medium.

The second experiment of this study was performed in order to overcome the problems identified in the first assay. The adaptation methodology was similar, with the following changes: cell cultures were initiated in 25 cm^2^ T-flasks, and only three of the initial five combinations of supplements were tested.

## Results

In the first experiment, cells growing in medium supplemented with the two combinations containing a trace element in common died at 2.5 % serum, while for the remaining combinations cell death occurred at a later stage, 0.625 % serum. Cell death was attributed to problems with the procedure of adaptation used in the first experiment, which were identified and corrected in the second experiment. The problems found and the procedure modifications implemented included the use of higher initial cell concentrations to allow the survival of an increased number of cells during the process; avoiding procedures that can be harsh to the cells, such as centrifugation and the use of enzymes (i.e. trypsin) due to a higher sensitivity of cells during adaptation; and allowing enough time in each step of the process for a complete cell adaptation.

After these modifications, in the second experiment, it was possible to observe that cells required a long time to adapt to each level of serum concentration, particularly below 0.625 %. At the level of 0.31% serum, cells start to detach, and become fully detached at 0.15 %, growing in suspension from this point on. The 0.31 % of serum was the most critical step of the process, demanding more time for cell adaptation and causing the death of cells growing in two of the combinations assayed. Indeed, only cells growing in medium supplemented with one of the combinations were able to survive the whole process.

It should also be noted that adaptation of cells to EX-CELL is easily achieved as long as some serum supplementation is maintained.

## Conclusions

This study demonstrated that the process of adaptation to serum-free medium is very challenging to the cells. They become extremely sensitive to common cell culture procedures, such as centrifugation or the use of enzymes, and consequently extra care should be taken when developing the adaptation procedure. Furthermore, it was shown that cell adaptation to serum-free conditions is affected by the medium supplements used, as well as the time given to each step of the process.

